# Functional characterization of *SOX5* variant causing Lamb–Shaffer syndrome and literature review of variants in the *SOX5* gene

**DOI:** 10.1186/s13023-025-03829-7

**Published:** 2025-06-11

**Authors:** Ping Wang, Hanbing Xie, Xiao Xiao, He Wang, Yan Wang, Shanling Liu

**Affiliations:** 1 Department of Medical Genetics/Prenatal Diagnostic Center, West China Second University Hospital, Sichuan University, Chengdu, Sichuan China; 2 Key Laboratory of Birth Defects and Related Diseases of Women and Children (Sichuan University), Ministry of Education, Chengdu, Sichuan China; 3 Obesity and Metabolism Medicine-Engineering Integration Laboratory, Department of General Surgery, The Third People’s Hospital of Chengdu, Affiliated Hospital of Southwest Jiaotong University, Chengdu, Sichuan China

**Keywords:** Lamb–Shaffer syndrome, Trio-whole-exome sequencing, SRY-box transcription factor 5 gene, Functional analysis

## Abstract

**Background:**

Lamb–Shaffer syndrome (LAMSHF, OMIM 616803) is a neurodevelopmental disorder caused by mutations in the SRY-box transcription factor 5 (*SOX5*) gene. The SOX5 protein is a conserved transcription factor with a high-mobility-group domain that enhances the expression of various extracellular matrix genes by promoting SOX9 binding to a distant enhancer of the target gene.

**Methods:**

We reported a 7-year-old boy with severe intellectual disability, seizures, autism, strabismus, and myopia, who carries a novel *SOX5* gene variant (c.1769T > C, p.Leu590Ser) inherited from his mother, who has a milder phenotype. We conducted in vitro assays to evaluate the effects of this variant and performed a literature review to explore the clinical and genetic spectrum of LAMSHF.

**Results:**

In silico and in vitro data suggest that the *SOX5* missense variant (c.1769T > C, p.Leu590Ser) may be pathogenic due to reduced transcriptional activation activity. Common characteristics of LAMSHF include intellectual disability, language delay, hypotonia, strabismus, autism spectrum disorder, seizures, and dysmorphic facial features. Although no clear genotype-phenotype association was found in LAMSHF, variable expressivity was noted.

**Conclusions:**

Our findings expand the genetic spectrum of LAMSHF and highlight the intrafamilial variability in severity among affected individuals. This study provides a comprehensive overview of the clinical manifestations of LAMSHF, aiding in diagnosis and genetic counseling.

**Supplementary Information:**

The online version contains supplementary material available at 10.1186/s13023-025-03829-7.

## Introduction

Lamb–Shaffer syndrome (LAMSHF, OMIM 616803) is a neurodevelopmental disorder caused by mutations in the SRY-box transcription factor 5(*SOX5*) gene [1]. LAMSHF is clinically characterized by global developmental delay, intellectual disability, expressive language delay, behavioral disturbances, and mild dysmorphic facial features [2]. The dysmorphic features include downslanting palpebral fissures, strabismus, frontal bossing, crowded teeth, and auricular abnormalities. The *SOX5* gene is located in the 12p12.1 region and produces several major transcript isoforms due to differential splicing. The longest isoform (NM_006940.6, also known as *L-SOX5A*) encodes a 763-amino-acid protein that is highly expressed in various tissues, including cartilage, brain and muscle [3, 4]. In contrast, the shortest isoform (NM_178010.4) encodes a 377-amino-acid protein specifically expressed in the testis [5].

The SOX (SRY-related HMG-box) protein family comprises a conserved group of transcription factors characterized by a high-mobility-group (HMG) domain [6]. This HMG domain, composed of three α-helices, binds to the 5’-AACAAT-3’ or related motifs [7]. SOX proteins are integral to various developmental processes, including sex determination, neurogenesis, and skeletogenesis [8]. The 20 SOX proteins are classified into eight groups (SOXA to SOXH) based on sequence identity within and outside the HMG domain [9, 10]. The SOXD group, which includes SOX5, SOX6 and SOX13, has a family-specific HMG domain in the C-terminal region and two coiled-coil domains in the N-terminal half. These coiled-coil domains mediate dimerization and efficient binding to adjacent HMG DNA sites [11]. SOX5 promotes the expression of many extracellular matrix genes in chondrocytes, such as aggrecan (*Acan*) and *COL11A2*, by enhancing the binding of SOX9 to an upstream enhancer of the target gene [12, 13]. Additionally, the combination of SOX5 and SOX9 can activate the *Acan* or *COL11A2* gene in non-chondrocytic cells following transient transfection with SOX expression plasmids [14].

In this study, we reported a novel variant of the *SOX5* gene found in a 7-year-old boy and his mother, which is likely disease-causing. Our findings revealed varying severities among patients within the same family who share the identical *SOX5* missense variant. RNA sequencing results elucidated the transcriptional status of HEK293 cells transfected with wild-type (WT) and variant plasmids. Furthermore, we conducted in vitro functional assays to assess the impact of the variant.

## Materials and methods

### Trio whole-exome sequencing (Trio-WES)

Genomic DNA was extracted from peripheral blood samples of the proband and his parents and analyzed using trio whole-exome sequencing (Trio-WES). Exome target enrichment was performed using the Nano WES Human Exome V2 (Berry Genomics). Paired-end sequencing was conducted on the Illumina NovaSeq6000 platform, generating 150-bp reads. The reads were aligned to the GRCh38/hg38 reference genome using Burrows-Wheeler Aligner software. Variants were annotated with ANNOVAR and the Enliven Variants Annotation Interpretation System (Berry Genomics). To filter out low-frequent variations, we utilized the gnomAD (http://gnomad.broadinstitute.org/) and the 1000 Genomes Project (1000G, http://browser.1000genomes.org). Scientific literature and disease databases, including PubMed (https://www.ncbi.nlm.nih.gov/pubmed/), ClinVar (http://www.ncbi.nlm.nih.gov/clinvar), OMIM (http://www.omim.org), the Human Gene Mutation Database (HGMD, http://www.hgmd.org), and the Human Genome Variation Society (http://www.hgvs.org/dblist/dblist.html), were applied to evaluate the pathogenicity of single nucleotide variants. The classification of variants followed the American College of Medical Genetics and Genomics (ACMG) guidelines (Richards et al., 2015).

The candidate variant was validated by Sanger sequencing in both the proband and his parents. The primer pairs were as follows: Forward-5′- CAGAAGAAGCCTCAATGATG-3′ and Reverse-5′-AGGAAGTGCTGGAGTCTC-3′.

### Protein-DNA docking

The DNA sequence contains a consensus binding site for HMG box proteins (5’-ACACTGAGAACAAAGCGCTCTCACAC-3’ and 5’-GTGTGAGAGCGCTTTGTTCTCAGTGT-3’) [15]. The structure of DNA was prediction using supercomputing facility for bioinformatics & computational biology webserver (http://www.scfbio-iitd.res.in/software/drugdesign/bdna.jsp). The 69-amino-acid DNA-bound HMG domain (position: 556–624) of the SOX5 protein was regarded as the active sites. Protein-DNA docking of SOX5 (UniProt ID: P35711) with DNA was performed using the HDOCK server (http://hdock.phys.hust.edu.cn/). In this analysis, DNA was considered as a ligand, while the SOX5 protein served as the receptor. The interactions between the SOX5 protein and DNA were visualized using the Discovery Studio Visualizer. The model with the lowest docking energy score was selected for a detailed analysis of the interactions between the molecules.

### Subcloning of *SOX5* cDNA

Human *SOX5* cDNA (NM_006940.6, 2292 bp) with a Flag tag at the N-terminus and EGFP was cloned into the pRP[Exp]-EGFP/Puro-EF1A > 3xFLAG/*hSOX5* vector (VectorBuilder, China). *SOX5* cDNA variants were generated through site-directed mutagenesis polymerase chain reaction (PCR) using a Gold Medal mix (#TSE102; Tsingke Biotechnology, China). The c.1769T > C (p.Leu590Ser) *SOX5* variant was generated using the following primer pairs: Forward-5′- CAAGATATCGGGATCTCGCTGGAAAGCT-3′ and Reverse-5′-ATCCCGATATCTTGCTGATGTTGGAGTTG-3′. The PCR products were digested with DpnI restriction endonuclease (#R0176V, New England Biolabs, USA) and then introduced into TOP10 Chemically Competent Cell (#TSC-C12, Tsingke, China) to obtain positive clones. Constructs of the WT and c.1769T > C (p.Leu590Ser) variant of the *SOX5* gene were verified by Sanger sequencing.

### Cell culture and transfection

Human embryonic kidney 293 (HEK293) cells were obtained from the Chinese Academy of Sciences Cell Bank (GNHu-43). HEK293 cells were cultured in DMEM (#11965092, Gibco, USA) supplemented with 10% fetal bovine serum (FBS, #SFBS-X, Bovogen, AUS), 100U/mL penicillin, and 100 µg/mL streptomycin (#C0222, Beyotime Biotechnology, China) in a humidified incubator at 37 °C with 5% CO_2_. For transient transfection, HEK293 cells were transfected with 4 µg of human *SOX5*(WT or variant) expressing plasmids per 6-well cell plate using 10 µL of PEI Transfection Reagent (#HY-K2014, MedChemExpress, USA) according to the manufacturer’s instructions. Adjustments to the plasmid DNA and PEI Transfection Reagent were made for different plate specifications.

### Immunofluorescence staining

HEK293 cells were transfected as described above and stained 24 h post-transfection. The cells were fixed with 4% paraformaldehyde, blocked with 5% bovine serum albumin and 0.3% Triton X-100, and then incubated overnight at 4 °C with an anti-Flag tag mouse antibody (1:400, #66008-3-Ig, Proteintech, USA). After being washed with phosphate-buffered saline (PBS), the cells were incubated for 1 h at room temperature with a Cy™3 AffiniPure™ Donkey Anti-Mouse IgG (H + L) antibody (1:600, #715-165-150, Jackson Immunoresearch, USA), followed by treatment with 4,6-diamidino-2-phenylindole (DAPI, 1:5000, #C1002, Beyotime Biotechnology, China). Fluorescent images were acquired using a Stellaris 5 confocal microscope (Leica, Germany).

### Western blotting

Cytoplasmic proteins were extracted using a Nuclear and Cytoplasmic Protein Extraction Kit (#P0027, Beyotime Biotechnology, China), after which the nuclear pellets were lysed with lysis buffer (#P0013, Beyotime Biotechnology, China). Protein lysates were then separated by 10% odium dodecyl sulfate–polyacrylamide gel electrophoresis (SDS-PAGE) and transferred onto a PVDF membrane. The membranes were blocked with 5% nonfat dried milk and incubated with an anti-Flag tag mouse antibody (1:4000, #F1804, Sigma, USA), followed by HRP-conjugated Goat Anti-mouse IgG (H + L) (1:10000, #SA00001-1, Proteintech, China). GAPDH was used as a loading control for cytoplasmic proteins, while Histone H3 served as the loading control for nuclear fraction proteins. Finally, proteins were visualized using chemiluminescence (WBKLS0500, Millipore), and the membranes were exposed to X-ray film in a darkroom.

### Reporter assay

HEK-293 cells were transfected in a 24-well cell plate with increasing amounts (0, 150, 300 ng) of *SOX5* WT or variant, 50 ng of the *SOX9* expression plasmid (pRP[Exp]-Puro-EF1A > FLAG/h*SOX9*[NM_000346.4]), 500 ng of the *ACAN* [4xA1]-Luc reporter, and 100 ng of the Renilla luciferase reporter (pRP[Exp]-EF1A > Rluc), as previously described [12]. Luciferase activities were measured using the Dual Luciferase reporter assay kit (# RG027, Beyotime Biotechnology, China) 48 h post-transfection. The luciferase activity was expressed as firefly luciferase normalized to transfection efficiency, using Renilla luciferase as a reference.

### RNA-sequencing (RNA-seq)

To analyze the differential gene expression profiles in HEK293 cells transfected with *SOX5* WT and variant plasmids, we divided the cells into distinct groups: three samples transfected with *SOX5* WT plasmids served as the control group, while three samples transfected with *SOX5* variant plasmids constituted the experimental group. After transfecting the cells with the plasmids for 24 h, we added 1 µg/ml of puromycin. Following an additional 24-hour incubation, samples were collected for sequencing. Total RNA was extracted, and sequencing libraries were constructed. These experiments were conducted by Gene Denovo Biotechnology Co. (Guangzhou, China). Differential expression of RNAs was analyzed using DESeq2 software to compare the two groups.

### Statistical analysis

Statistical analyses were conducted using SPSS Statistics 24.0 (SPSS Inc., USA). Data are presented as mean ± standard deviations (SD). Differences between groups were assessed using a two-tailed Student’s t-test. *P*-values < 0.05 were considered statistically significant.

## Results

### Clinical description

**Fig. 1:**
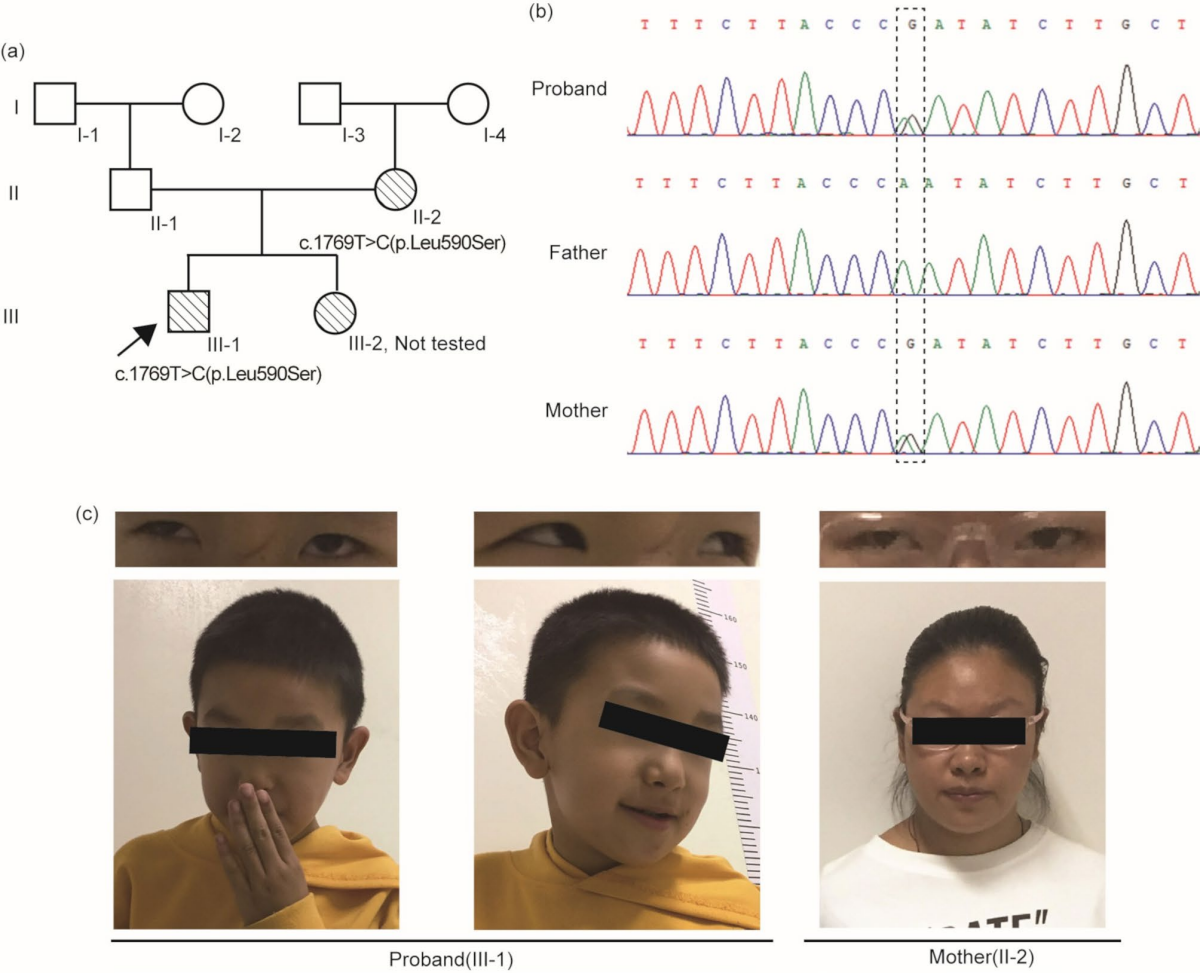
Clinical characteristics of the family. (**a**) Pedigree of the analyzed family in this study. A missense variant (c.1769T > C, p.Leu590Ser) in the *SOX5* gene was detected in both the proband and his mother, with the proband indicated by an arrow. (**b**) Sanger sequences results of the *SOX5* gene within the family. (**c**) Facial features of the proband and his affected mother, both carrying the *SOX5* variant (c.1769T > C, p.Leu590Ser)

A 7-year-old boy, born to a non-consanguineous Chinese couple, was referred to West China Second University Hospital, Sichuan University (Chengdu, China). His mother had irregular prenatal examinations, but he was delivered at term. Following his birth, the proband exhibited significant developmental delays. He experienced his first seizure at the age of 2 years, which was triggered by a fever. An electroencephalogram revealed abnormalities, but magnetic resonance imaging of the brain showed no significant findings. He subsequently began taking antiepileptic drugs and responded well to treatment. At 4 years old, he was diagnosed with autism, and his intelligence quotient was assessed at 46 points. During the latest evaluation at age of 7 years, he was able to speak in full sentences with simple structures, walk independently, and exhibited physical features such as a low nasal bridge, hypertelorism, strabismus and myopia (Fig. 1c).

The proband’s mother has been diagnosed with mild intellectual disability, autism, strabismus and myopia. The proband has a 5-year-old sister who exhibits slightly lower memory capabilities, although this has not been quantified. The father shows no related clinical manifestations. The family pedigree is illustrated in Fig. 1a.

### Genetic findings

**Fig. 2:**
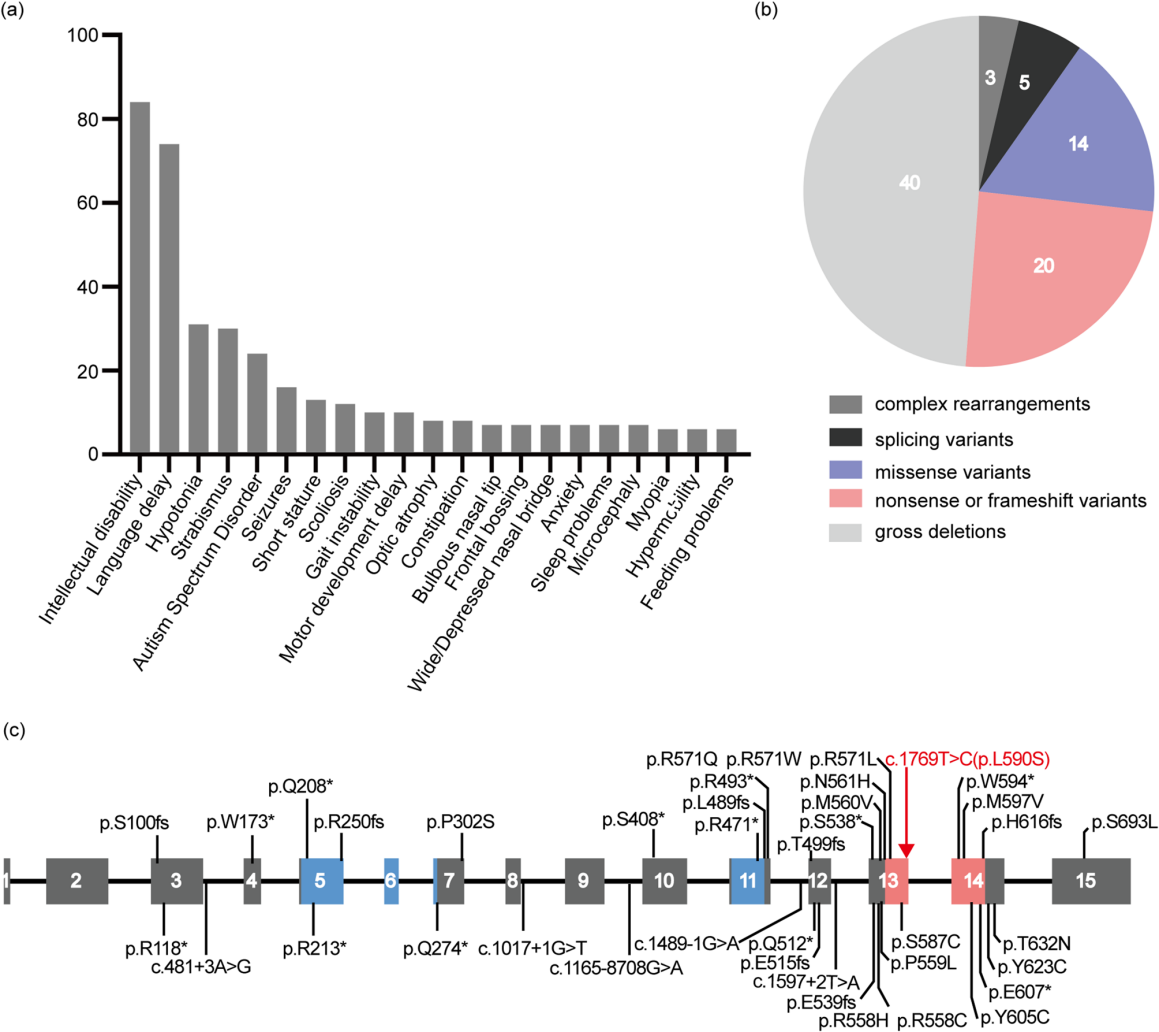
Clinical manifestations of Lamb–Shaffer syndrome in the literature. (**a**) The frequency of various clinical manifestations of Lamb–Shaffer syndrome is summarized based on existing literature and our study. (**b**) The distribution of *SOX5* variant types in the Human Gene Mutation database. (**c**) The location of *SOX5* variants reported in the present study (in red) and those identified previously (in black) are shown on a schematic model of the *SOX5* gene. Coding regions are represented by grey boxes, while introns are depicted as lines. The functional domains are indicated: the coiled-coil domain (blue) and the high-mobility-group domain (pink)

A novel heterozygous variant, NM_006940.6: c.1769T > C (GRCh38/hg38: g.23543213T > C), in the *SOX5* gene (OMIM: 604975) was identified in the proband and his mother, but was not present in his father. The results were further confirmed by Sanger sequencing (Fig. 1b). This missense variant is highly conserved across species and results in the substitution of leucine with serine. The c.1769T > C (p.Leu590Ser) variant is located in exon13 and the HMG domain of the SOX5 protein (Fig. 2c). According to ACMG criteria, the variant was classified as likely pathogenic (PP3_S, PM2_P, PM1).

### In silico predictions

**Fig. 3:**
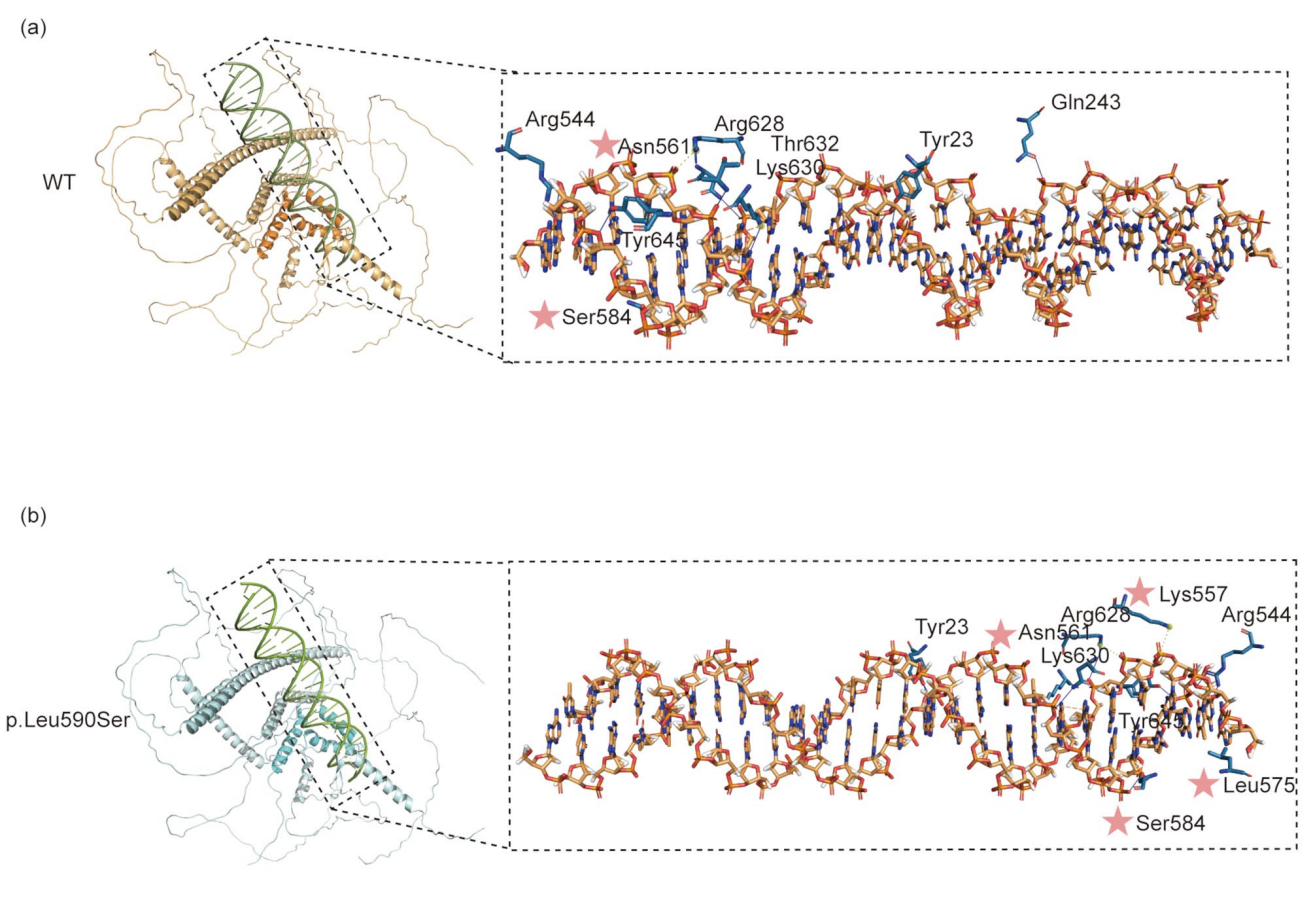
Molecular docking between SOX5 and DNA. Protein-DNA docking was performed using the HDOCK server. The high-mobility-group domain, spanning position 556–624 of both SOX5 wild type (WT) and p.Leu590Ser proteins, is highlighted in a darker color. In the binding sites of the SOX5 protein-DNA interaction, the pink star denotes the site located within the HMG domain (positions 556–624) of the SOX5 protein

To investigate whether the interaction between *SOX5* variants and DNA was altered, we carried out protein-DNA docking. The HDOCK server indicated that the SOX5 WT protein interacts with the DNA molecule through ten hydrogen bonds involving Tyr23, Tyr23, Gln243, Arg544, Arg544, Asn561, Ser584, Lys630, Thr632 and Tyr645, as well as one π-cation interaction with lys630 and one salt bridge with Arg628. In contrast, the SOX5 p.Leu590Ser variant interacts with DNA through nine hydrogen bonds involving Tyr23, Tyr23, Arg544, Arg544, Asn561, Ser584, Arg628, Lys630 and Tyr645, one hydrophobic bond with Leu575, one π-cation interaction with lys630, and two salt bridges with Lys557 and Arg628. Notably, among the active molecules forming hydrogen bonds with DNA, the SOX5 variants Asn561 and Ser584 are consistent with the WT. Additionally, the SOX5 variant also formed a salt bridge with Lys557 and a hydrophobic bond with Leu575 (Fig. 3). These results suggest that the theoretical interactions of the SOX5 variant protein with DNA may differ from those of the WT. A summary of the predictions made by the HDOCK server is presented in Table 1.

**Table 1:** The interactions in protein–DNA molecular Docking between SOX5 protein and DNA using the HDOCK server

Ligand	Receptor	Number of Hydrogen Bonds	Amino Acids with Hydrogen Interactions	Amino Acids with π-cation interaction	Amino Acids with salt bridge	Amino Acids with hydrophobic bond
DNA (a consensus binding site for HMG box proteins)	Wild type	ten	23Tyr, 23Tyr, 243Gln, 544Arg, 544Arg, **561Asn**, **584Ser**, 630Lys, 632Thr, 645Tyr	630lys	628Arg	/
	p.Leu590Ser	nine	23Tyr, 23Tyr, 544Arg, 544Arg, **561Asn**, **584Ser**, 628Arg, 630Lys, 645Tyr	630lys	**557Lys**, 628Arg	**575Leu**

HMG: high-mobility-group; DNA sequence: 5’-ACACTGAGAACAAAGCGCTCTCACAC-3’ and 5’-GTGTGAGAGCGCTTTGTTCTCAGTGT-3’

### *SOX5* variant (c.1769T > C) does not affect protein subcellular localization or expression levels

**Fig. 4:**
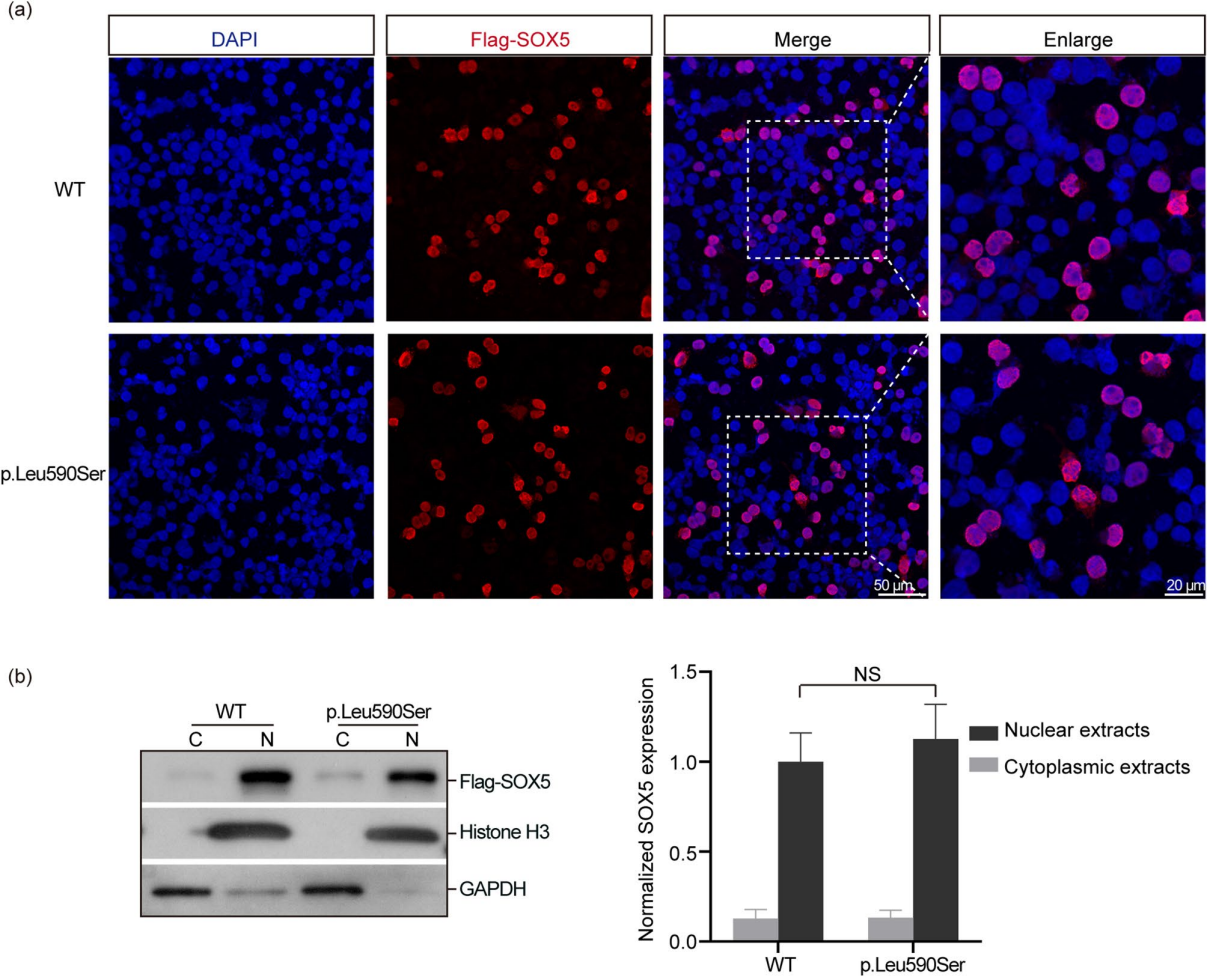
*SOX5* variant does not affect subcellular localization or expression level of its protein. (**a**) Immunostaining of human embryonic kidney 293 (HEK293) cells transfected with either *SOX5* wild-type (WT) or its variants. Both WT and variant were expressed in the nuclei. Nuclei were visualized using DAPI. (**b**) Western blotting analysis of SOX5 expression in HEK293 cells. The expression level in the SOX5 WT and variants was no statistically significant difference. The expression of SOX5 protein in the nuclear fraction was normalized to Histone H3, while the cytoplasmic protein was normalized to GAPDH. C: cytoplasmic extracts; N: nuclear extracts; NS: no significant

We generated the *SOX5* c.1769T > C variant using PCR site-directed mutagenesis to assess its functional impact on HEK293 cells, and the sequences were verified by Sanger sequencing. To investigate the subcellular distribution directly, HEK293 cells transfected with either *SOX5* WT or the c.1769T > C variant were stained with an anti-Flag-tag antibody that specifically recognizes the exogenous SOX5 protein. The SOX5 WT protein primarily localized in the nucleus, while the SOX5 p.Leu590Ser protein was also able to translocate into the nucleus (Fig. 4a). This result was anticipated, as the variant is located far from the N-terminal nuclear import signal. Western blots of nuclear and cytoplasmic fractions demonstrated that both SOX5 WT and p.Leu590Ser protein were predominantly found in the nuclear fraction. Furthermore, there was no statistically significant difference in expression levels between the SOX5 WT and p.Leu590Ser protein in HEK293 cells (*P* > 0.05, Fig. 4b). Transfection efficiencies were nearly identical between cells transfected with *SOX5* WT and the variant (Supplementary Fig. 1).

### *SOX5* variant (c.1769T > C) inhibits SOX5’s role in transactivation

**Fig. 5:**
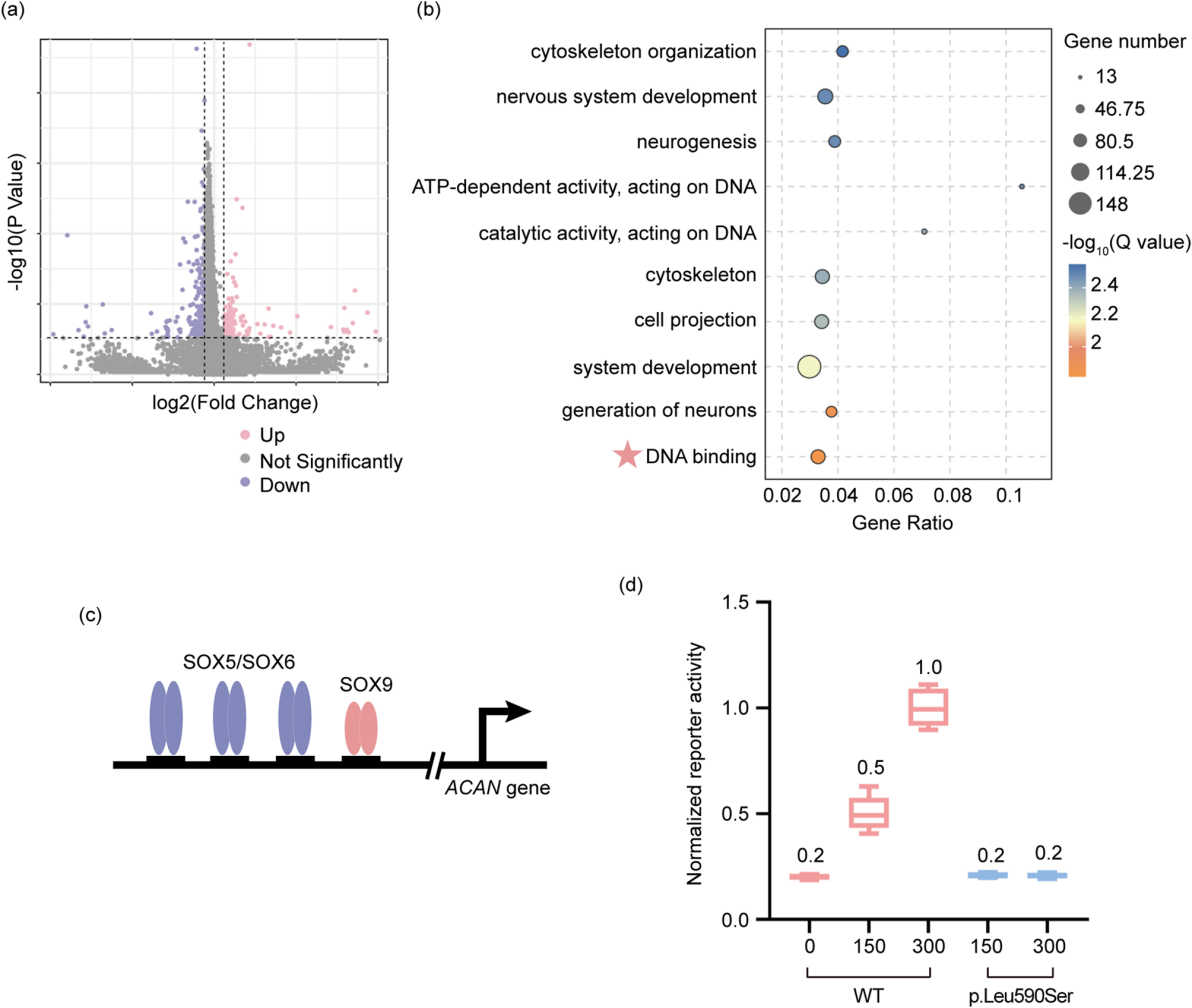
*SOX5* variant (p.Leu590Ser) decreased transcriptional activation activity. (**a**) This comparison revealed a set of differentially expressed genes in human embryonic kidney 293 (HEK293) cells transfected with either wild type (WT) or variant plasmid, as depicted in the volcano plots. (**b**) Gene Ontology analysis of the differentially expressed genes in HEK293 cells transfected with the *SOX5* variant, compared to WT. (**c**) A proposed model for the transactivation of *ACAN* by the SOX trio. SOX9 dimers require SOXD (SOX5/6) dimers, which bind to DNA sites within the *ACAN* enhancer. (**d**) HEK-293 cells were transfected with *ACAN* and Renilla luciferase reporter plasmids, along with plasmids encoding SOX9 and SOX5. The *SOX5* WT or variant (p.Leu590Ser) plasmids were used in indicated amounts (0, 150, 300 ng). 0ng represents the empty vector control. Luciferase activity was normalized to that of the *SOX5* WT plasmids at 300 ng

To further elucidate the functional impact of the *SOX5* variant (c.1769T > C) on cells, we employed RNA-seq to identify differentially expressed genes in HEK293 cells. Compared to the WT, 238 genes were upregulated and 208 genes were downregulated in HEK293 cells transfected with the *SOX5* variant (c.1769T > C), based on total RNA analysis with a *P* value of ≤ 0.05 and a fold change ≥ 1.5 (Fig. 5a). Subsequent Gene Ontology (GO) analysis revealed that HEK293 cells transfected with the *SOX5* variant (c.1769T > C) were enrichment in pathways related to cytoskeleton organization, nervous system development, neurogenesis, and DNA binding, among others. (Fig. 5b, false discovery rate, FDR < 0.05). This enrichment suggests that the *SOX5* variant (c.1769T > C) may affect the binding affinity of the SOX5 protein to DNA, thereby influencing its transcriptional activation activity on downstream target genes. Further cellular functional experiments are needed to validate the results obtained from the RNA-seq analysis.

SOX5/SOX6 and SOX9 form a trio of transcription factors essential for upregulating the *ACAN* gene and other cartilage-related genes necessary for chondrogenesis (Fig. 5c) [16]. To assess the transcriptional activity of the *SOX5* variants, we transfected HEK293 cells with expression plasmids for *SOX5* WT or the variant, *SOX9*, *ACAN* reporters, and Renilla luciferase reporters. *SOX5* WT enhanced the transcriptional activation activity of *ACAN* in a dose-dependent manner. In contrast, the *SOX5* c.1769T > C variant reduced transcriptional activation activity by 79.15% at 150 ng and by 79.22% at 300 ng (*P* < 0.001). Furthermore, the transcriptional activation level of the SOX5 c.1769T > C variant was comparable to that of the blank control (Fig. 5d).

### Literature revision on patients carrying a *SOX5* variant

To enhance clinicians’ recognition of LAMSHF, we evaluated the frequency of various clinical manifestations associated with this disease. Figure 2a illustrated the prevalence of each clinical feature reported in patients with LAMSHF. The most common characteristics included intellectual disability, language delay, hypotonia, strabismus, autism spectrum disorder, seizures, and dysmorphic facial features. Notable facial features encompassed a bulbous nasal tip, frontal bossing, a depressed or wide nasal bridge, prominent philtral ridges and epicanthal folds. While no clear genotype-phenotype association was observed in LAMSHF, variable expressivity was noted. The clinical manifestations were summarized based on their association with respective variants (Supplementary Table 1).

As of January 2024, a total of 82 pathogenic variants of the *SOX5* gene associated with LAMSHF have been documented in the HGMD (Fig. 2c). Excluding three complex rearrangements (3/82, 3.66%) and forty gross deletions (40/82, 48.78%), there were twenty nonsense or frameshift variants (20/82, 24.39%), fourteen missense variants (14/82, 17.07%), and five splicing variants (5/82, 6.10%, Fig. 2b). Truncating variants (i.e., nonsense, splice site, and frameshift variants) were located within or before the HMG domain, resulting in a loss of DNA-binding capability. Additionally, these variants may induce nonsense-mediated mRNA decay, thus contributing to LAMSHF. The majority of missense variants occur in the HMG domain, with four located in the first coiled-coil domain and three in the second coiled-coils domain. It was suggested that these pathogenic variants might disrupt DNA binding or protein dimerization.

## Discussion

In this study, we presented clinical, genetic, bioinformatics, and functional analyses demonstrating that the novel *SOX5* gene variant (c.1769T > C, p.Leu590Ser) causes LAMSHF.

This research significantly enhances our understanding of the highly variable expressivity of LAMSHF, even among family members carrying the same *SOX5* variant. The proband exhibited severe intellectual developmental delay, epilepsy (controllable with antiepileptic medications), autism, strabismus, and myopia. In contrast, his mother, who carried the identical SOX5 variant, displayed only mild intellectual disability, strabismus, and myopia, with no signs of epilepsy or autism. The sister, who declined genetic testing, showed only slight memory decline. The variability in phenotype severity among family members may be attributed to differing genetic backgrounds and environmental factors. Further experiments are needed to confirm the specific mechanisms underlying these highly variable phenotypes.

Previous studies have shown that the HMG domain mediates DNA binding and plays a crucial role in the function of *SOX5*. The variant (c.1769T > C, p.Leu590Ser) identified in this study is located within the HMG domain. It is known that the SOX5 WT is expressed in the cell nucleus [17–19]. Compared to HEK293 cells transfected with the *SOX5* WT plasmid, there were no changes in subcellular localization or expression levels when HEK293 cells were transfected with the variant (c.1769T > C, p.Leu590Ser). Additionally, there was no statistically significant difference in transfection efficiency between the two groups, indicating that the observed consistency was not due to differences in plasmid transfection efficiency.

After performing RNA-seq on HEK293 cells transfected with *SOX5* WT and variant (c.1769T > C, p.Leu590Ser) plasmids, we focused on analyzing the top 30 GO terms with significant differences based on differentially expressed genes. We observed that the differentially expressed genes were mainly enriched in terms related to generation of neurons, DNA binding, nervous system development, and neurogenesis. SOXD proteins (SOX5/6) interacts with SOX9 to regulate many extracellular matrix genes (e.g., *Acan* and *Col11a2*) [20, 21]. Subsequently, we conducted dual-luciferase reporter assays to verify whether the *SOX5* variant reduced transcriptional activation of the *ACAN* gene. Compared to HEK293 cells transfected with *SOX5* WT plasmids, those transfected with the *SOX5* variant (c.1769T > C, p.Leu590Ser), *SOX9* expression plasmids, *ACAN* reporters, and Renilla luciferase reporters exhibited decreased *ACAN* expression. This is consistent with the report by Zawerton et al., which indicated that missense mutations in the HMG domain of the SOX5 protein reduce its ability to activate transcription of downstream genes [22]. After transfecting the *SOX5* variants into HEK293 cells, the variants still exhibited transcriptional activation activity on target genes, and the activation levels showed no statistically significant difference compared to the blank control. This confirms that SOX9 is essential for the transcription of extracellular matrix genes (e.g., *Acan*, *COL11A2*), while SOX5/SOX6 can promote the transcriptional activation of target genes [12].

Most patients with LAMSHF have *de novo* gross deletions involving all or part of the *SOX5* gene, while a few have truncating variants (i.e., nonsense, splice site, and frameshift variants) or missense variants [23, 24]. All truncating variants are distributed from the N-terminus to the HMG domain of the SOX5 protein. These variants may lead to nonsense-mediated mRNA decay or loss of the HMG domain (if nonsense-mediated decay does not occur), ultimately resulting in LAMSHF. Here, we reported a patient with LAMSHF harboring a novel missense variant (c.1769T > C, p.Leu590Ser) inherited from his affected mother, thereby expanding the mutational spectrum of the pathogenic *SOX5* gene associated with LAMSHF. Previously, most of reported cases were sporadic, with proband carrying *de novo* mutations. This report was a familial case. This study may offer insights into genetic counseling of this disease.

## Conclusion

In conclusion, our study extends the genetic spectrum of missense variant occurring in the HMG domain of the *SOX5* gene. The study provides varying severity among patients within a family who carried the identical *SOX5* missense variant. Both in silico and in vitro functional data indicate that the *SOX5* novel missense variant (c.1769T > C, p.Leu590Ser) is pathogenic due to decreasing transcriptional activation activity. This study provides deeper support for genetic counseling and prenatal diagnosis in LAMSHF patients.

## Electronic supplementary material

Below is the link to the electronic supplementary material.

**Supplementary Material 1**: **Supplementary Fig. 1** There were negligible differences in transfection efficiencies between human embryonic kidney 293 (HEK293) transfected with either *SOX5* wild-type (WT) or its variant.

Supplementary Material 2

## Data Availability

The data and materials that support the findings of this study are available from the corresponding author upon reasonable request.

## Abbreviations

LAMSHF: Lamb-Shaffer syndrome
SOX5: SRY-box transcription factor 5
WT: Wild-type
HMG: High-mobility–group
PCR: Polymerase chain reaction
