# Correction: Functional characterization of *SOX5* variant causing Lamb–Shaffer syndrome and literature review of variants in the *SOX5* gene

**DOI:** 10.1186/s13023-025-04032-4

**Published:** 2025-09-25

**Authors:** Ping Wang, Hanbing Xie, Xiao Xiao, He Wang, Yan Wang, Shanling Liu

**Affiliations:** 1 Department of Medical Genetics/Prenatal Diagnostic Center, West China Second University Hospital, Sichuan University, Chengdu, Sichuan China; 2 Key Laboratory of Birth Defects and Related Diseases of Women and Children, Sichuan University, Ministry of Education, Chengdu, Sichuan China; 3 Obesity and Metabolism Medicine-Engineering Integration Laboratory, Department of General Surgery, The Third People’s Hospital of Chengdu, Affiliated Hospital of Southwest Jiaotong University, Chengdu, Sichuan China

**Correction: Orphanet Journal of Rare Diseases (2025) 20:300**

10.1186/s13023-025-03829-7

Following publication of the original article [1], the authors reported an error in Fig. 1. It was noticed that the figure in the downloadable article PDF unintentionally compromised the anonymity of both individuals.

The correct Fig. 1 is:


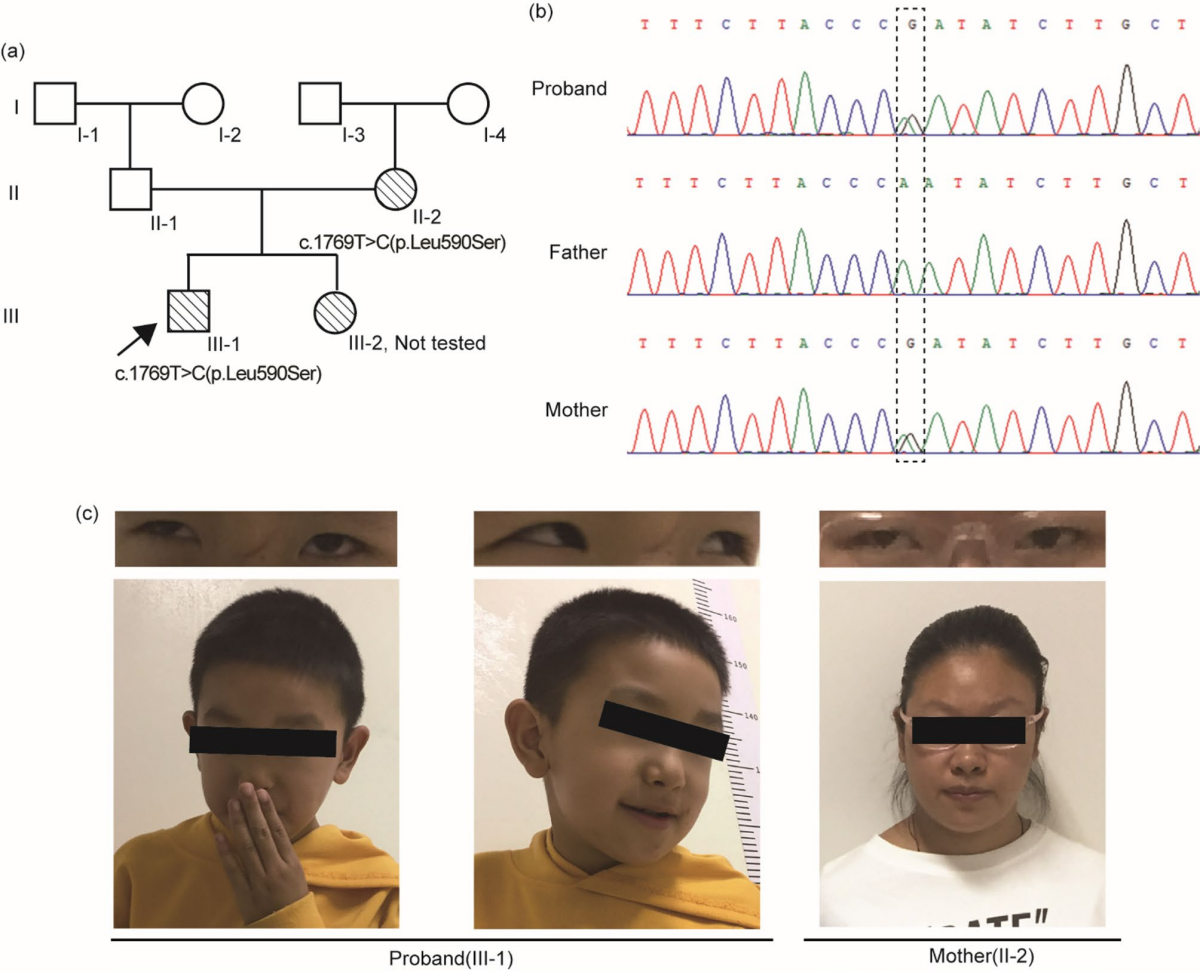


The original article [1] has been updated.
