# Supplementary figures and images for: Functional characterization of SOX5 variant causing Lamb–Shaffer syndrome and literature review of variants in the SOX5 gene
Source: Orphanet J Rare Dis. 2025 Jun 11;20:300. doi: 10.1186/s13023-025-03829-7 (PMC12160102; doi:10.1186/s13023-025-03829-7)

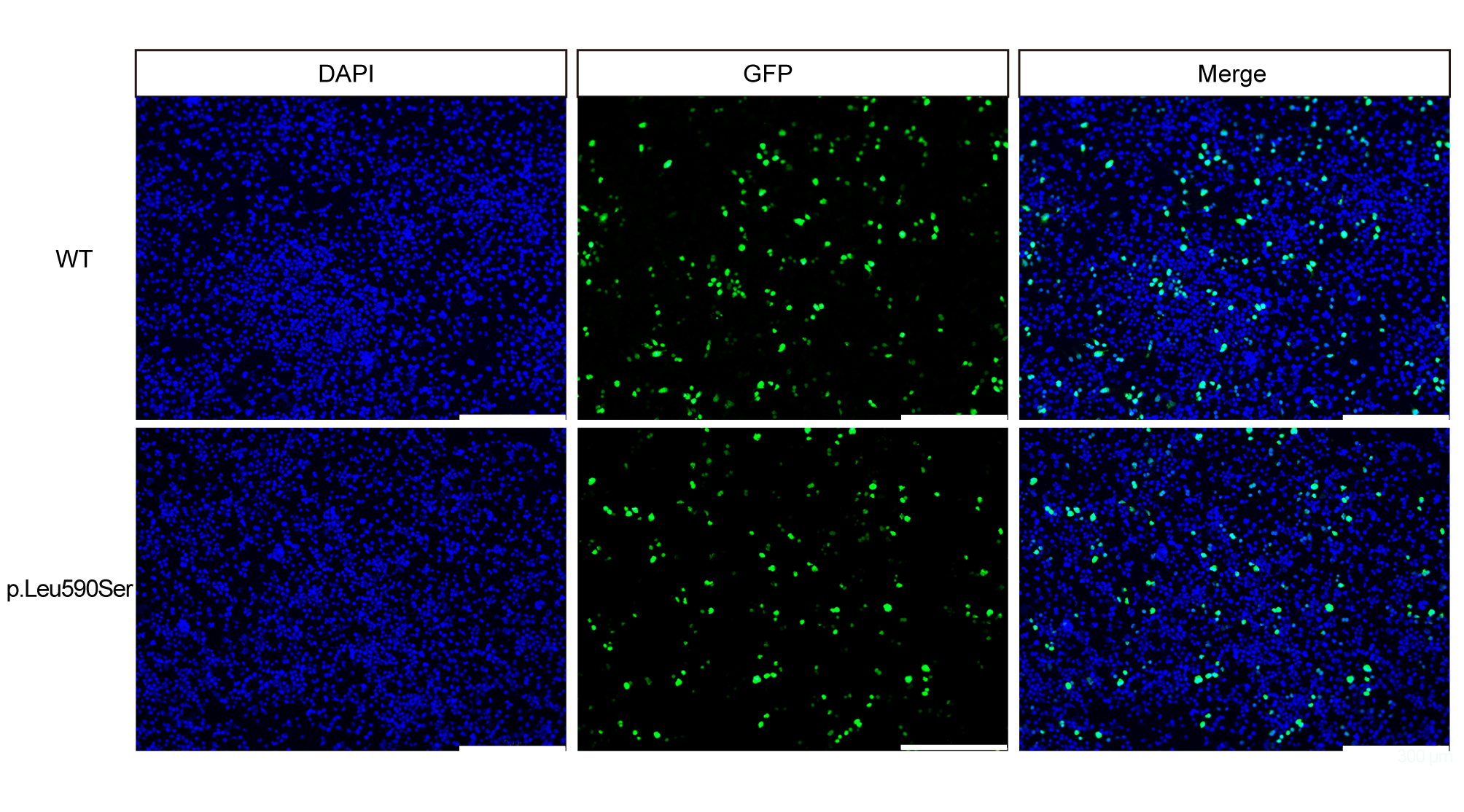

Supplement: Supplementary file 1 — Supplementary Material 1: Supplementary Fig. 1 There were negligible differences in transfection efficiencies between human embryonic kidney 293 (HEK293) transfected with either SOX5 wild-type (WT) or its variant. [file 13023_2025_3829_MOESM1_ESM.tif]
